# Neurologic adverse events of cancer immunotherapy

**DOI:** 10.1590/0004-282X-ANP-2022-S116

**Published:** 2022-08-12

**Authors:** Marcelo Houat de Brito

**Affiliations:** 1 Universidade de São Paulo, Hospital das Clinicas, Departamento de Neurologia, São Paulo SP, Brazil.; 2 Universidade de São Paulo, Instituto do Câncer do Estado de São Paulo, Departamento de Neurologia, Sao Paulo SP, Brazil.

**Keywords:** Immune Checkpoint Inhibitors, Immunotherapy, Adoptive, Drug-Related Side Effects and Adverse Reactions, Neurologic Manifestations, Neuromuscular Diseases, Inibidores de Checkpoint Imunológico, Imunoterapia Adotiva, Efeitos Colaterais e Reações Adversas Relacionados a Medicamentos, Manifestações Neurológicas, Doenças Neuromusculares

## Abstract

Cancer immunotherapy encompasses a wide range of treatment modalities that harness the anti-tumor effects of the immune system and have revolutionized oncological treatment in recent years, with approval for its use in more and more cancers. However, it is not without side effects. Several neurological adverse events have been recognized associated with immune checkpoint inhibitors (ICI) and chimeric antigen receptor (CAR) T-cell therapy, the two main classes of cancer immunotherapy. With the increase in the prevalence of oncological diseases and this type of therapy, it is improbable that neurologists, oncologists, hematologists, and other healthcare professionals who deal with cancer patients will not encounter this type of neurologic complication in their practice in the following years. This article aims to review the epidemiology, clinical manifestations, diagnosis, and management of neurological complications associated with ICI and CAR T-cell therapy.

## RESUMO

A imunoterapia contra o câncer engloba uma gama de modalidades de tratamento que aumentam os efeitos antitumorais do próprio sistema imunológico do paciente e revolucionaram o tratamento oncológico nos últimos anos, com aprovação para seu uso em cada vez mais neoplasias. No entanto, não é sem efeitos colaterais. Vários eventos adversos neurológicos foram reconhecidos associados aos inibidores de checkpoint imunológico (ICI) e à terapia de células T com receptor de antígeno quimérico (CAR-T), as duas principais classes de imunoterapia contra o câncer. Com o aumento da prevalência de doenças oncológicas e desse tipo de terapia, é improvável que neurologistas, oncologistas, hematologistas e demais profissionais de saúde que lidam com pacientes com câncer não encontrem esse tipo de complicação neurológica em sua prática nos próximos anos. Este artigo tem como objetivo revisar a epidemiologia, as manifestações clínicas, o diagnóstico e o manejo das complicações neurológicas associadas à terapia com ICI e células CAR-T

## INTRODUCTION

In recent decades, we have seen a demographic and epidemiological transition globally, generating population aging and an increase in the incidence and prevalence of non-communicable chronic diseases, such as cancer. These result in a significant increase in years lived with disability due to oncologic pathologies, estimated at 40.6% between 2007 and 2017^1^. Another important factor that has led to the increase in the prevalence of oncological diseases has been the evolution of their treatment, with the emergence of new classes of therapies, such as cancer immunotherapy.

Cancer immunotherapy encompasses a wide range of treatment modalities that harness the anti-tumor effects of the immune system. Some immunotherapies broadly activate the immune system while others target precisely distinct tumor antigens^2^. This modality has revolutionized oncological treatment in recent years, with approval for its use in more and more cancers, changing their prognosis. However, it is not without side effects, some of them potentially serious. Several neurological adverse events have been recognized associated with these novel immunotherapeutic concepts^3^. With the increase in the prevalence of oncological diseases and this type of therapy, it is improbable that neurologists, oncologists, hematologists, and other healthcare professionals who deal with cancer patients will not encounter this type of neurologic complication in their practice in the years that follow.

This article will review the epidemiology, clinical manifestations, diagnosis, and management of neurological complications associated with immune checkpoint inhibitors (ICI) and chimeric antigen receptor (CAR) T-cell therapy, two of the main classes of cancer immunotherapy.

## IMMUNE CHECKPOINT INHIBITORS (ICI)

Immune checkpoint inhibitors are a class of antineoplastic drugs that enhance antitumor immune responses through the upregulation of T-cell activity. They are specific monoclonal antibodies that block receptors that inhibit the T-cell response, the so-called inhibitory immune checkpoints. The main targets of these medications are cytotoxic T lymphocyte antigen 4 (CTLA-4) receptor, programmed cell death 1 (PD-1) receptor, and programmed cell death 1 ligand (PD-L1), which are molecules that ultimately break the T-cell immune-mediated response^4^. Their blockage has led to persistent and generalized activation of the humoral and cellular adaptative immune system, enhancing antitumor immunity^5^.

There are currently seven ICIs approved for clinical use: the anti-CTLA-4 ipilimumab; the anti-PD-1 pembrolizumab, nivolumab, and cemiplimab; and the anti-PD-L1 atezolizumab, avelumab, and durvalumab^6^. They have shown clinically effective antitumor response and improved survival for melanoma, non-small cell lung cancer (NSCLC), renal cell carcinoma, as well as for an increasing number of other indications. However, because of their effect in activating the immune system, they are associated with immune-related adverse events (irAE). The most common irAEs are reactions involving the gastrointestinal tract, endocrine glands, skin, and liver^7^. Most of these are mild and can be treated with symptomatic medications, but some require interruption or discontinuation of the ICI and the use of IV steroids or other immunosuppressive drugs (e.g., infliximab for colitis)^8^. 

Although less frequent than other systems, neurologic irAEs (nirAE) may be severe and require prompt recognition and treatment^9^ ^,^ ^10^. A 2019 pharmacovigilance study from the Japanese Adverse Drug Event Report database found a 7.67% incidence of any nirAE in patients who used ICI^11^. In a real-life study, with data from over 1,800 patients undergoing ICI therapy, the frequency of severe (Common Terminology Criteria for Adverse Events grade 3-5) nirAE was 2.2% among patients treated with CTLA-4 inhibitors, 1.0% among patients receiving PD-1/PD-L1 inhibitors, and 2.8% among patients receiving combined treatment with drugs targeting the PD-1 and CTLA-4 pathways^12^. As seen in previous statistics, anti-CTLA4 was more associated with nirAE than other ICIs, with this risk being greater the higher the dose, or if use is associated with anti-PD1, this also increases the chance of more severe symptoms^13^ ^,^ ^14^. NirAE appears to be more frequent in patients with melanoma than other cancers^10^ ^,^ ^15^. There seems to be no difference between sex and age when comparing patients who used ICI and had neurological complications with those who did not^15^. Symptom onset is most frequent in the first three to four months after ICI initiation, although it may occur at any time during the treatment^3^.

Among the proposed pathophysiological mechanisms for the emergence of irAE is (1) a shift toward the pro-inflammatory profile of T lymphocytes dominated by Th1/Th17 differentiation that increases the production of pro-inflammatory cytokines, (2) autoreactive antibody production, (3) activation of potentially pre-existing self-reactive T cells, and (4) cross-reactivity between normal tissue antigens and tumor neo-antigens^16^. There seem to be cases of both patients who start to develop autoimmune phenomena and cases of exacerbation of already-present clinically manifested or latent autoimmunity, since the documentation of worsening of immune-mediated neurological diseases after the use of ICI (e.g., multiple sclerosis relapse), as well as the presence of autoantibodies associated with immune-mediated neurological diseases in several cases of nirAE (e.g., presence of antibodies related to a paraneoplastic neurological syndrome)^3^ ^,^ ^10^ ^,^ ^12^ ^,^ ^16^.

A recent systematic review gathered the cases of nirAE present in publications, verifying the most frequent forms of presentation. Myositis (32%) was the most frequent neurological complication, followed by peripheral neuropathies (22%), myasthenic syndrome (14%), encephalitis (13%), cranial neuropathy (7%), central nervous system (CNS) demyelinating disease/myelopathy ( 4%), and aseptic meningitis (3%)^10^. From now on, we will review the particularities of each of these clinical presentations and cite other less frequent presentations already reported. Table 1 summarizes the main forms of nirAEs' epidemiology, clinical manifestations and diagnostic workup.

**Table 1.** Main forms of immune checkpoint inhibitor-associated neurologic adverse events.

Syndrome	% of nirAE^10^	Clinical manifestations	Diagnostic workup	Mortality rate^10^
Myositis	32%	-Proximal muscular weakness with myalgia -Ptosis, dysphagia -Facial and neck weakness -Respiratory dysfunction -Myocarditis	-↑ serum CK and aldolase -EMG: myopathic pattern -Ab: SM, AChr (not frequent) -Muscle biopsy: lymphocyte infiltration -MNM, EKG, Echo: assess concomitant myocarditis	17%
Myasthenic syndromes	14%	-Ocular myasthenia -Generalized myasthenia -Myositis / myocarditis overlap -LEMS	-NCS with repetitive nerve stimulation -EMG with single-fiber evaluation -Ice pack test (when ptosis is present) -Ab: AChr, VGCC	28%
Peripheral neuropathy	22%	-Acute polyradiculoneuropathy (GBS-like) -CIDP -Sensory neuronopathy -Miller-Fisher syndrome -Others: Phrenic neuropathy, vasculitic neuropathy, small fiber neuropathy, enteric neuropathy, neuralgic amyotrophy, motor neuropathy, Mononeuritis multiplex	-NCS -CSF: albuminocytologic dissociation or pleocytosis with high protein -Ab: rarely positive, GM1 more common -MRI: contrast enhancement in nerve roots, plexus, and/or cranial nerves in polyradiculoneuropathy	11%
Cranial neuropathy	7%	-Facial nerve palsy -Vestibulocochlear nerve impairment -Trigeminal nerve impairment -Oculomotor nerve palsy. -Multiple cranial neuropathy simultaneously	-CSF: albuminocytologic dissociation or pleocytosis with high protein -MRI: contrast enhancement in affected cranial nerves -NCS (facial palsy)	0%
Encephalitis	13%	-Altered mental status, cognitive impairment, seizures, psychiatric disturbances and/or movement disorders	-MRI: Mesial temporal lobes, basal ganglia, cortico-subcortical, and/or brainstem areas of increased signal in T2/FLAIR -CSF: mild pleocytosis, elevated protein, OCB+ -EEG: diffuse slow activity; epileptic or slow-wave activity involving focal cerebral regions -Ab (CSF and serum): Ma2, Hu, others	21%
CNS demyelination / myelopathy	3,5%	-MS relapse -NMOSD -Isolated optic neuritis -Isolated myelitis -Atypical demyelination	-MRI: periventricular, juxtacortical, infratentorial, spinal cord, and/or optic nerve involvement characteristic of MS or NMOSD -CSF: mild pleocytosis, elevated protein, OCB+ -Ab: AQP4 (NMOSD)	12%
Aseptic meningitis	3%	- headache, neck stiffness, fever and/or nausea	-MRI: meningeal contrast enhancement -CSF: mild pleocytosis and elevated protein	0%

nirAE: neurologic immune-related adverse events; CK: creatine kinase; EMG: electromyography; Ab: antibody; SM: striatal muscle; AChr: acetylcholine receptor; MNM: myocardial necrosis markers; EKG: electrocardiogram; Echo: echocardiogram; LEMS: Lambert-Eaton myasthenic syndrome; NCS: nerve conduction study; VGCC: voltage-gated calcium channel; GBS: Guillain-Barré syndrome; CIDP: chronic inflammatory demyelinating polyneuropathy; CSF: cerebrospinal fluid; GM1: ganglioside-monosialic acid; MRI: magnetic resonance imaging; FLAIR: fluid-attenuated inversion recovery OCB: oligoclonal bands; EEG: electroencephalogram; MS: multiple sclerosis; NMOSD: neuromyelitis optica spectrum disorder; AQP4: aquaporin 4

### Myositis

The most frequent form of nirAE can range from increases in creatine kinase (CK) with few symptoms to severe, life-threatening cases, such as respiratory muscle involvement or necrotizing myopathy evolving with rhabdomyolysis^3^ ^,^ ^17^. The most common presentation is a limb-girdle pattern of muscular weakness associated with myalgia involving predominantly proximal muscles. The involvement of ocular muscles, mainly ptosis, and bulbar muscles are also frequent. Facial and neck muscle involvement, respiratory dysfunction, and myocarditis are more common in ICI-associated myopathy than in other inflammatory myopathies, such as polymyositis or dermatomyositis^10^. Myocarditis can occur in up to 32% of cases of ICI-related myositis, and it is important to perform cardiologic evaluation in patients with this condition, including myocardial enzymes, electrocardiogram, and echocardiogram^17^ ^,^ ^18^. 

In addition to increases in CK, the diagnostic workup may find a pattern of myopathy on electromyography (EMG) and muscle edema and other findings compatible with myositis on muscle magnetic resonance imaging (MRI)^10^ ^,^ ^17^. Histological analyses of muscle biopsy typically demonstrate infiltration of the muscle tissue with lymphocytes^3^. Antibodies (Ab) associated with myositis are found in just over a third of cases, the most common being anti-striated muscle (SM), followed by anti-acetylcholine receptor (AChr)^10^. 

Most patients received steroid treatment, which in some cases, especially in the refractory ones, had the addition of other immunomodulatory therapies, such as intravenous immunoglobulin (IVIG), plasma exchange (PLEX), azathioprine, mycophenolate, tacrolimus, infliximab, and cyclosporine^10^ ^,^ ^12^ ^,^ ^19^. Partial or complete improvement is seen in the majority of the cases. However, a mortality rate of 17% has been reported, mainly due to respiratory failure and sudden cardiac arrest associated with myocarditis^10^.

### Myasthenic syndrome

A characteristic of the myasthenic syndromes associated with ICI is the tendency to severity. The most common clinical presentation is generalized myasthenia, with respiratory failure and bulbar muscle involvement in most cases. The isolated ocular form was identified in less than 20% of patients^10^. It is commonly associated with myositis, and myocarditis may also be present, adding morbidity to the condition^10^ ^,^ ^17^ ^,^ ^18^. In contrast to conventional myasthenia gravis (MG), where around 85% of anti-AChr Ab positivity and 90% of any Ab positivity exists^20^, only approximately 60% of ICI-related myasthenia patients described had Ab positivity, with anti-AChr representing its totality^3^. There is also a case report of Lambert-Eaton Myasthenic Syndrome (LEMS) associated with nivolumab in a patient with pulmonary squamous cell carcinoma and positive anti-P/Q-type voltage-gated calcium channel (VGCC) Ab^21^. 

Similar to non-ICI-related myasthenic syndromes, Nerve conduction study (NCS), including repetitive nerve stimulation, and single-fiber electromyography could be used to document neuromuscular junction compromise. EMG is also helpful in evaluating the presence of concomitant myositis, as are the CK serum levels^12^ ^,^ ^18^. The ice pack test in cases with ptosis also seems to be very useful for diagnosis in myasthenic syndromes associated with ICI, as well as in classic MG^18^ ^,^ ^22^ ^,^ ^23^. 

Most reported patients were treated with pyridostigmine and steroids associated with another immunomodulatory treatment, including IVIG, PLEX, rituximab, and/or mycophenolate^3^ ^,^ ^10^ ^,^ ^17^. Although most patients showed a favorable response to treatment and relapses were uncommon, the mortality rate was 28% in ICI-induced myasthenia cases described, being the highest mortality rate of a nirAE^10^. Respiratory failure was the most frequently reported cause of death^10^.

### Peripheral neuropathy

Several forms of peripheral neuropathy have been reported associated with ICI^10^ ^,^ ^17^ ^-^ ^19^. The most frequent condition was acute or subacute demyelination polyradiculoneuropathy, with sensory and/or motor deficits primarily affecting the extremities, typically symmetrical, areflexia, and cranial nerve impairment in some cases, similar to Guillain-Barré syndrome (GBS)^3^ ^,^ ^10^. Other presentations described were sensory neuropathy/neuronopathy, chronic inflammatory demyelinating polyradiculoneuropathy, plexopathy, Miller-Fisher syndrome, phrenic neuropathy, vasculitic neuropathy, small fiber neuropathy, isolated enteric neuropathy, neuralgic amyotrophy, motor neuropathy, and mononeuritis multiplex^10^. Severe dysautonomia may also occur in cases of ICI-related peripheral neuropathy^10^ ^,^ ^24^ ^,^ ^25^. 

NCS could confirm peripheral nerve impairment and demonstrate the pattern of injury, whether axonal or demyelinating^3^ ^,^ ^18^. Cerebrospinal fluid (CSF) analysis has abnormal findings in most cases, demonstrating albuminocytologic dissociation in almost half and an increase of both protein and cell count in about a third^10^. Autoantibody positivity was verified in just under a quarter of the peripheral neuropathy patients in whom they were measured, with GM1 being the only one verified in two different cases described in a nirAE systematic review^10^. In complex cases, in which the remainder of the diagnostic workup was doubtful, an MRI could help by demonstrating contrast enhancement in nerve roots, plexus, and/or cranial nerves in polyradiculoneuropathy as a nirAE^3^ ^,^ ^10^. 

Unlike classical GBS, in which corticosteroids have no evidence of benefit^26^, the treatment of peripheral neuropathies associated with ICI seems to have a great response to this therapy, even in cases of acute polyradiculoneuritis^3^ ^,^ ^10^ ^,^ ^27^. IVIG or PLEX are often used in severe or refractory cases^3^ ^,^ ^27^. Treatment with tacrolimus, rituximab, infliximab, and mycophenolate has also been reported^10^. The vast majority of patients with this condition had good responses to treatment. However, there is a reported mortality of 11% and described relapses following ICI rechallenge or after the end of neurologic therapy^10^.

### Cranial neuropathy

Isolated cranial neuropathies are less frequent than other forms of peripheral neuropathies. However, they still represent a considerable portion of the nirAE and are important in the differential diagnosis of myasthenic syndromes with facial involvement. In order of frequency, the most affected nerves are facial, optic, vestibulocochlear, trigeminal, and oculomotor. Although less common, the presentation could be bilateral and with multiple cranial nerves simultaneously^10^.

CSF examination is usually altered in most cases, and there may be an overlap with asseptic meningitis and hypophysitis. MRI may demonstrate contrast enhancement in the affected nerve. The treatment usually follows the same principles described in peripheral neuropathies, with most cases having an excellent response to corticosteroid therapy, despite some reports of recurrence during weaning^10^ ^,^ ^12^ ^,^ ^28^.

### Encephalitis

Encephalitis is the main form of nirAE in the CNS, being potentially very serious, with a mortality rate of 21% among the described cases. Altered mental status, cognitive impairment, seizures, psychiatric disturbances and movement disorders are the primary presentation signs in order of frequency^10^. The condition seems to be directly triggered by ICI or an accelerated form of a paraneoplastic syndrome typically associated with the treated tumor (as in small-cell lung cancer)^5^ ^,^ ^29^. The latter is reinforced by about half of the reported cases having autoantibody positivity, with Ma2 and Hu Abs being the most frequent^10^. There are reports of two cases in which Ma2 Abs were positive even before treatment with ICI and then developed clinical encephalitis^30^ ^,^ ^31^.

As with non-ICI-related autoimmune encephalitis, MRI may be normal or demonstrate different patterns of brain involvement^3^ ^,^ ^10^ ^,^ ^29^ ^,^ ^32^. Mesial temporal lobes, basal ganglia, cortico-subcortical, and brainstem areas of increased signal in T2/FLAIR MRI have been described^10^ ^,^ ^33^. In most reported cases, CSF analysis demonstrated pleocytosis (mean 17 leukocytes/μL) and increased protein (mean 85mg/dL)^10^. Oligoclonal bands (OCB) were encountered in almost a quarter of the patients^10^. It is essential to discard infectious differential diagnoses in the CSF, such as HSV encephalitis^29^ ^,^ ^32^. Another important complementary test is the electroencephalogram (EEG), which may demonstrate epileptic or slow-wave activity involving focal cerebral regions. The mesial temporal lobe involvement on EEG is present in the proposed diagnostic criteria for autoimmune limbic encephalitis, the most common form of ICI-related encephalitis^5^ ^,^ ^10^ ^,^ ^32^.

Most cases were treated with corticosteroids in association with another method, including IVIG, PLEX, rituximab, cyclophosphamide, and natalizumab^10^. Symptomatic treatment of epileptic seizures, movement disorders, dysautonomia, sleep, and behavioral symptoms may be required together with immunomodulatory treatment and still more in the case of sequelae^34^. Despite the high reported mortality rate, most patients still have a complete or partial response to treatment^10^.

### CNS demyelination and myelopathy

A 2020 systematic review specifically evaluated central nervous system demyelination associated with ICI^16^. Five patients with multiple sclerosis (MS) were described, with two distinct patterns: three patients already diagnosed with the disease who had a relapse during ICI use, and two patients with radiologically isolated syndrome (i.e., with demyelinating lesions highly suggestive of MS but without clinical symptoms of the disease)^35^ who developed symptoms after the use of ICI and then fulfilled criteria for the diagnosis of MS^36^. One reported patient met the criteria for neuromyelitis optica spectrum disease (NMOSD) by developing longitudinally extensive transverse myelitis (LETM) after exposure to nivolumab, with documentation of the presence of anti-AQP4 Ab^37^. Another similar case was described after this systematic review, with pembrolizumab-induced LETM and positive anti-AQP4^38^. Seven cases of myelitis, four cases of isolated optic neuritis, and six cases of what was called atypical demyelination were also reported in the 2020 systematic review; none of these met the criteria for the two earlier-mentioned diseases.

In the diagnostic workup, brain, spine, and optic nerves MRI may demonstrate typical findings for MS or NMOSD. CSF could show modest pleocytosis, increased protein content, and presence of OCB^10^ ^,^ ^16^. Most patients were treated with corticosteroids. PLEX, IVIG, cyclophosphamide, mycophenolate, and infliximab were also used^16^. The last of these is interesting since the potential of TNF-α blockers in triggering or aggravating demyelination is known^39^. Infliximab is already used more widely in ICI-related refractory colitis with good outcomes^40^, suggesting that decreasing the pro-inflammatory state associated with TNF-α helps treat irAE. The two patients who used infliximab had isolated myelitis refractory to steroids and PLEX or IVIG, with improved neurological symptoms with the anti-TNF-α^16^. The reported patients diagnosed with MS (demyelinating pathology defined) and ICI-associated relapse used corticosteroids in the acute phase and interferon or glatiramer as a disease-modifying drug^16^. None of them used infliximab; therefore the effects in this specific group could not be better evaluated. 

### Aseptic meningitis

Its most common clinical manifestations are headache, neck stiffness, fever and nausea^3^. Patients with melanoma and treated with ipilimumab are more likely to develop aseptic meningitis than carriers of other neoplasms that have used another ICI^10^. The key diagnostic findings are sterile CSF with lymphocytosis and brain MRI demonstrating meningeal contrast enhancement^3^. The prognosis is usually excellent, probably the nirAE having the best percentage of response to therapy, which is performed mainly with corticosteroids alone^10^ ^,^ ^12^.

### Other CNS syndromes

There are descriptions of six cases of subacute cerebellar degeneration related to ICI^10^, a syndrome with a known paraneoplastic association^41^. Despite this, antibodies were negative in the cases tested. Half of the patients had MRI abnormalities, including cerebellar edema, T2/FLAIR hyperintensity lesions, and contrast enhancement. CSF usually demonstrates mild pleocytosis and increased protein^10^.

Other CNS reported syndromes associated with ICI were posterior reversible encephalopathy syndrome (PRES, n=5), neurosarcoidosis (n = 2), CNS vasculitis (n = 2), opsoclonus myoclonus, leptomeningitis with cranial nerves involvement, steroid-responsive encephalopathy associated with autoimmune thyroiditis, mild encephalitis with reversible splenial lesion, neuro-Sjögren syndrome, non-defined CNS granulomatosis, Tolosa-Hunt syndrome, orbital inflammatory syndrome, bilateral internuclear ophthalmoplegia, and isolated akathisia^10^.

Although best characterized as an endocrinologic adverse event, hypophysitis is often reported as a nirAE^3^ ^,^ ^11^. This probably occurs because of the anatomical proximity, symptoms similar to those manifested in some neurological syndromes, and the likelihood of simultaneous neurological involvement (e.g., aseptic meningitis). Hypophysitis or isolated hypopituitarism were found in 2.45% of patients undergoing treatment with ICI present in the Japanese Adverse Drug Event Report database, which was more frequent than the other groups of neurological syndromes reported^11^. This AE is typically grade 1/2 in severity and often presents non-specific symptoms, including fatigue, muscular weakness, and headaches, making it challenging to diagnose^42^. Diagnostic workup includes serum hormonal levels and MRI to evaluate the function and integrity of the pituitary gland and exclude differential diagnoses such as tumor metastasis or pituitary apoplexy^3^. The condition is treated with the replacement of deficient hormones only, as systemic high-dose corticosteroids do not appear to be beneficial^43^.

### Management of nirAE

There is a European Society for Medical Oncology (ESMO) guideline for managing immunotherapy toxicities that tries to provide guidance based on the best available evidence for the treatment of nirAE^8^. Unfortunately, the evidence for neurological syndromes is low, considered level V, based on studies without a control group, case reports, or expert opinions. This guideline recommends checkpoint inhibitor therapy be withheld until the nature of the AE is defined, except for mild (grade 1) neurological symptoms. Also, except for mild cases, corticosteroids are recommended; prednisolone 0.5mg/kg/day orally for moderate cases (grade 2) and prednisolone 1-2mg/kg/day orally or equivalent intravenously for more severe cases (grades 3 and 4). The possible necessity for use of IVIG and PLEX is specifically mentioned in GBS and myasthenic syndrome cases. It is important to point out that other therapies have already been described in publications and previously mentioned in this article (i.e., cyclophosphamide, rituximab, infliximab). They could be used in cases refractory to the guideline-recommended therapies. The oral corticosteroids could be tapered down within four to eight weeks depending on symptom severity^23^. Table 2 summarizes nirAE treatment recommendations.

**Table 2.** Management of immune checkpoint inhibitor-associated neurologic adverse events.

Grade of neurologic toxicity (CTCAE)	Management
I - Mild symptoms	-Continue ICI -Neurologic vigilance
II - Moderate symptoms, limiting instrumental ADL	-Delay ICI -Low dose steroids^a^ (prednisolone 0,5mg/kg/d)
III or IV - Severe symptoms, limiting self-care ADL (III), life threatening (IV)	-Discontinue ICI -High dose steroids^a^ (prednisolone 1-2mg/kg/d or IV equivalent) -Consider IVIG or PLEX -Refractory cases: consider other immunomodulatory therapies^b^ (i.e., rituximab, cyclophosphamide, infliximab)

a oral corticosteroids could be tapered down within 4-8 weeks depending on symptom severity; ^b^: limited evidence, based on individual case reports of each specific neurologic syndrome; CTCAE: common terminology criteria for adverse events; ICI: immune checkpoint inhibitor; ADL: activities of daily living; IVIG: intravenous immunoglobulin; PLEX: plasma exchange.

An adequate diagnostic investigation should be delivered in every suspected case of nirAE. This could avoid potentially serious differential diagnoses being left untreated (i.e., herpetic encephalitis, meningeal carcinomatosis) and unnecessary ICI discontinuation. It is also essential to keep in mind that the treatment not only involves the immunomodulatory part. Treatment of specific symptoms of each syndrome could be needed, such as using pyridostigmine in myasthenic syndromes, antiepileptic drugs when seizures occur, or ventilatory support in neuromuscular syndromes with respiratory failure. Therefore, for better differential diagnosis of neurological symptoms and specific symptomatic treatment, the ESMO guideline advises a neurologist's early evaluation of nirAE-suspected cases^8^.

### ICI rechallenge

As a general rule, guidelines on the subject do not recommend ICI rechallenge after cases of severe nirAE^8^ ^,^ ^44^. However, there are cases where there is no other effective alternative for cancer treatment or where ICI treatment had a fantastic response previously, and the disease recurred with its discontinuation. In this context, many oncologists, neurologists, and patients accept the risks of nirAE relapse and opt for cautious retreatment with ICI therapy to manage advanced malignancy. There are few data in the literature to inform risk or provide data-driven recommendations for ICI rechallenge after severe nirAE. A 2020 case series reported ten patients who had a severe nirAE and were re-treated with ICI, demonstrating a 60% recurrence rate^12^. Half of those patients either did not receive immunosuppressive therapy to manage the initial event or received a short course of oral prednisone (less than two weeks). Therefore, it is likely that the recurrence rate among ICI re-treated patients could be reduced by treating all severe nirAE with immunosuppressive therapies^12^.

## CAR T-CELL THERAPY

Chimeric antigen receptors (CAR) are engineered receptors that graft a defined specificity onto an immune effector cell, typically a T cell, and augment T-cell function. One of the main problems with the body's immune response against cancer is that tumor antigens are often shared with healthy tissues. Mechanisms to avoid autoimmunity end up mitigating the antitumor response, making it often transient or ineffective. The rationale of CAR T-cell therapy is to overcome this immune tolerance^45^. It is done by collecting the patient's own T cells, modifying them with a CAR transgene targeting tumor antigens, expanding the cells, and reinfusing them into the patient after preconditioning chemotherapy (usually with fludarabine and cyclophosphamide)^46^. After infusion, CAR T cells leave the blood and travel to sites of the tumor, where they identify and kill tumor cells. This can trigger extensive proliferation of CAR T cells and the release of tumor antigens, which activates the immune system to recruit non-CAR T cells, thus eliciting further antitumor responses in a process known as cross priming^45^.

The first CAR T-cell therapy approved by the United States Food and Drug Administration (FDA) was tisagenlecleucel, targeting CD19 antigen^47^. It was first indicated against B-cell acute lymphoblastic leukemia (ALL) in children and young adults and was later accepted for B-cell non-Hodgkin lymphoma (NHL). As of March 2022, the FDA approved another five CAR T-cell therapies. Three are directed against CD19 to treat lymphomas and/or B-cell ALL in adults: axicabtagene ciloleucel, brexucabtagene autoleucel, and lysocabtagene maraleucel. The other two target B-cell maturation antigen (BCMA) for the treatment of multiple myeloma (MM): idecabtagene vileucel and ciltacabtagene autoleucel^48^. While this treatment is more and more frequently used against hematological malignancies in clinical routine, it needs to be seen if this approach will also work against solid tumors^3^. This therapy has shown excellent results, with impressive, long-lasting remission rates in patients with relapsed/refractory hematologic cancers. Although the clinical responses of these agents in these malignancies have been very encouraging, they have also produced substantial morbidity and occasionally mortality resulting from toxicity^49^. 

The most common form of CAR T-cell toxicity is the cytokine release syndrome (CRS), a supra-physiologic response following immune therapy that results in activation or engagement of endogenous or infused T cells^50^. Its incidence varies depending on the neoplasm and the therapy used; it may reach up to 100% with mild symptoms and up to 46% in the cases with grade 3 or greater symptoms^51^. CRS usually begins with fever, myalgia, rigors, and fatigue within the first one to 14 days following CAR T-cell infusion and can include hypotension, vascular leak, hypoxia, and/or end organ dysfunction. These manifestations may be progressive and can last two to three weeks, although this is often resolved sooner with optimal management^46^ ^,^ ^52^. CRS treatment is made with corticosteroids and tocilizumab, a monoclonal antibody that blocks the IL-6 receptor, one of the significantly elevated cytokines in patients with this syndrome. Siltuximab, which also acts on the IL-6 pathway, is another option that can be used^51^. Although it is not a directly neurological adverse event, it is important to know about CRS because there may be an overlap of part of its pathophysiology with that of the neurotoxicity associated with CAR T-cell, which will be better addressed from now on.

### Immune effector cell-associated neurotoxicity syndrome (ICANS)

Neurotoxicity associated with CAR T-cell therapy is known as immune effector cell-associated neurotoxicity syndrome (ICANS) and occurs with high frequency^3^ ^,^ ^46^ ^,^ ^49^ ^,^ ^52^. The incidence in studies with CD19-targeted CAR T-cell therapies ranges from 23%-67% for patients with lymphoma and 40%-62% for those with leukemia. About half of these cases are severe, grade 3 or more^49^. ICANS appears to be much less frequent in BCMA-targeted treatment of multiple myeloma and no toxic death due to ICANS has been reported in trials in patients with MM, but there are some reports of severe cases^53^. Similar neurotoxicity, usually grade 1 or 2, has also been reported using another type of cancer immunotherapy, the CD19/CD3-bispecific T-cell receptor-engaging antibody blinatumomab, used for relapsed/refractory ALL; this therapy is also related to CRS^3^ ^,^ ^46^ ^,^ ^54^.

CRS is more common than ICANS, and most of the patients with ICANS also present a CRS^55^. This leads us to think that the two conditions have a pathophysiological overlap, including previous studies demonstrating a relationship between the presence of ICANS and the severity of CRS^53^ ^,^ ^56^ ^,^ ^57^. As there are described cases of ICANS without CRS, it cannot be said that the pathophysiology of one is necessarily related to the other. Reports have suggested a role for IL-1 in pathophysiology of both CRS and ICANS; IL-6 does not seem to be directly related to ICANS since its blockade, one of the hallmarks of CRS treatment, does not decrease the incidence of neurotoxicity and may be linked to a slightly higher severe ICANS rate^46^ ^,^ ^58^. The main proposed mechanisms that lead to CAR T-cell neurotoxicity are endothelial activation and disruption of the blood-brain barrier integrity^3^ ^,^ ^56^. Myeloid cell activation in the CNS and high CSF levels of excitatory glutamate and quinolinic acid have already been documented in ICANS; the latter can be implicated in epileptogenesis^46^ ^,^ ^57^.

ICANS usually occurs within the first 28 days after the CAR T-cell infusion, often occurring during CRS or more commonly shortly after it ends^46^. Symptoms appear on average three days after the infusion and last for about two weeks^3^. Its typical presentation is similar to another toxic-metabolic encephalopathy, with lack of attention, confusion, myoclonus, and word-finding difficulty. Initially, symptoms may be mild, waxing and waning. However, they may progress in hours to a few days to more severe forms, such as global aphasia, seizures, motor weakness, diffuse cerebral edema, and coma^3^ ^,^ ^46^. Aphasia, ranging from mild fluency alteration to global aphasia with mutism, is perhaps the most specific symptom of this syndrome, which helps to differentiate it from other types of toxic-metabolic encephalopathy^46^ ^,^ ^57^. It is important to mention that high-grade ICANS was associated with bleeding and coagulation abnormalities, including prolonged prothrombin time, decreased fibrinogen, and increased d-dimer; it was also related to thrombosis, mainly deep vein thrombosis, but strokes have also been reported^59^.

Diagnostic workup is made through neurological examination including fundoscopy to exclude papilledema, EEG, neuroimaging, and lumbar puncture in some cases, in the absence of contraindications^53^ ^,^ ^60^. Although most EEG findings are nonspecific, such as diffuse slow activity, this test is important to rule out nonconvulsive status or subclinical seizures^46^. Neuroimaging, preferably MRI, is helpful in ruling out other acute neurologic abnormalities, such as ischemic or hemorrhagic stroke, and monitoring for signs of cerebral edema or the presence of underlying mass lesions. Most of the neuroimaging tests requested do not show anatomical alterations. However, MRI hyperintense signal abnormalities were already described on FLAIR and T2-weighted images secondary to vasogenic edema that may involve thalami, brainstem, basal ganglia, cingulate gyrus, hippocampus, and/or splenium of the corpus callosum; leptomeningeal enhancement and multifocal microhemorrhages have also been observed^61^. CSF analysis may be useful when there is a suspicion of neuroinfection or leptomeningeal disease progression, as well as measuring intracranial pressure (ICP). Ferritin and C-reactive protein, markers of inflammatory activity, can also be useful in the evaluation of these patients.

ICANS is usually self-limiting and completely reversible in most patients, although it is still uncertain whether it can cause long-term subclinical neurological sequelae. It is primarily managed with supportive care for low-grade toxicities and corticosteroids for more severe cases^46^. Care should be based on a multidisciplinary assessment since measures may be necessary to control behavior in a confused state (pharmacological and non-pharmacological), treatment of epileptic seizures and intracranial hypertension, as well as compensation for any other associated organic dysfunction.

In order to perform the grading of this condition similarly between services, helping to standardize clinical management, the American Society for Transplantation and Cellular Therapy (ASTCT) published an objective consensus grading system for ICANS^50^. An interesting instrument that is part of this graduation is the Immune Effector Cell-associated Encephalopathy (ICE) assessment tool (Table 3), which is somewhat similar to the mini-mental state exam (MMSE) but shorter and focused on the areas most affected by the disease. For children under 12 years of age, another tool, the Cornell Assessment of Pediatric Delirium (CAPD), was suggested^46,50^. Table 4 provides an adaptation of the ASTCT grading scale in conjunction with ICANS management guidelines^50^ ^,^ ^53^ ^,^ ^60^ ^,^ ^62^.

**Table 3.** Immune Effector Cell-Associated Encephalopathy (ICE) score to neurological toxicity assess^50^.

Test	Points
Orientation: orientation to year, month, city and hospital	4
Naming: name three objects (i.e., point to pen, clock and table)	3
Following commands: ability to follow simples commands (i.e., “smile”, “show me two fingers”	1
Writing: ability to write a standard sentence	1
Attention: ability to count backwards from 100 by 10	1

**Table 4.** The American Society for Transplantation and Cellular Therapy (ASTCT) grading system^50^ with respective management strategy for Immune effector cell-associated neurotoxicity syndrome (ICANS) - [adapted from Zhou, et al.^53^].

	Grade 1	Grade 2	Grade 3	Grade 4
ICE score	7-9	3-6	0-2	Unable to perform
Depressed level of consciousness	Awakens spontaneously	Awakens to voice	Awakens only to tactile stimulus	Unarousable or requires vigorous or repetitive tactile stimuli to arouse. Stupor or coma
Seizure	No	No	Any clinical seizure that resolves rapidly or non-convulsive seizures on EEG that resolve with intervention	Life-threatening prolonged seizure (>5 min) or repetitive clinical or electrical seizures without return to baseline in between
Motor findings	No	No	No	Deep focal motor weakness such as hemiparesis or paraparesis
Elevated ICP/cerebral edema	No	No	Focal/local edema on neuroimaging	Diffuse cerebral edema on neuroimaging; decerebrate or decorticate posturing; or cranial nerve VI palsy; or papilledema; or Cushing’s triad^a^
ICU	Alert ICU	Transfer to ICU	Transfer to ICU	Transfer to ICU
Alert neurologist, elevate the head of the patient’s bed to 30°, management of CRS if concurrent				
Management	Close monitoring	Dexamethasone IV 10mg every 6 h, and consider levetiracetam 750 mg bid as prophylaxis for seizures	Dexamethasone IV 20 mg every 6 h. If seizure, clonazepam IV 1mg or other benzodiazepines to terminate it, then loading with levetiracetam (or other available IV AED)	Management of seizure as per grade 3. If papilledema, start acetazolamide IV (or enteral if IV form not available) 1,000 mg followed by 250-1,000 mg bid. If elevated ICP/cerebral edema, consider hyperosmolar therapy with mannitol and hyperventilation. Methylprednisolone IV 1,000 mg/d. Evaluation of other experimental salvage options

a Irregular, decreased respirations, Bradycardia, Systolic hypertension; CRS: cytokine release syndrome; ICE: immune effector cell associated encephalopathy; ICP: intracranial pressure; ICU: intensive care unit; IV: intravenous; MRI: magnetic resonance imaging; AED: antiepileptic drug.

In conclusion, cancer immunotherapy has revolutionized oncological treatments, changing paradigms of neoplasms previously considered intractable due to refractoriness or poor prognosis. The trend is for new therapies to emerge involving immunotherapeutic concepts and expanding the indications of treatments already available for other neoplasms. However, it is crucial to keep in mind their adverse effects, especially the neurological ones, which are sometimes challenging to diagnose due to varied nonspecific symptoms and a broad differential diagnosis. Neurologists, oncologists, hematologists, and other healthcare professionals who deal with cancer patients should be aware and up-to-date regarding the neurological adverse events of immune checkpoint inhibitors and CAR T-cell therapy. They occur with considerable frequency, can be potentially serious, and should increasingly be seen in the coming years with a greater availability of these therapies**.**
